# The new human resources for health policy supports the need for South African family medicine training programmes to triple their output

**DOI:** 10.4102/safp.v63i1.5329

**Published:** 2021-05-26

**Authors:** Klaus B. von Pressentin

**Affiliations:** 1 Division of Family Medicine, School of Public Health and Family Medicine, Faculty of Health Sciences, University of Cape Town, Cape Town, South Africa

‘Building the future with family doctors’ is the 2021 World Family Doctor Day theme.^1^ But how does this theme align with our National Department of Health’s plans for the future of family medicine and primary care in South Africa? The much-awaited 2030 Human Resources for Health (HRH) policy represents a need for action for our discipline and the Department of Health, as highlighted in the accompanying strategic plan for 2020–2021 until 2024–2025.^2^ In the foreword, the Minister of Health highlighted:

[*T*]he need for government to take decisive steps to improve equity in the distribution of health care providers, between the public and private health sectors, and between urban and rural areas.^2^

This editorial wishes to draw the reader’s attention to the technical analyses which underpin the HRH policy’s planning and forecasting. The cause for concern is the limitations of the models used for this evaluation, specifically model II (health workforce needed for primary healthcare [PHC]) and model III (national need for specialist doctors). Model II described the PHC HRH needs and gaps but focused only on the primary level of care in the public sector and described an estimated gap of 154 full time equivalents (FTEs) of specialists by 2025. Detail on these medical specialists needed for PHC is missing. Model III aimed to project the supply and need for medical specialists in South Africa but focused only on the tertiary and quaternary levels of care (and not the district and secondary levels in the health system). The policy authors acknowledged these limitations, including model III’s assumption of a highly specialised service provision given that only a small percentage of healthcare visits requires highly specialised services and South Africa’s significant commitment to strengthen PHC.^3,4^

Table 1^2^ presents a subset of data from model III and aims to compare the projected supply for family medicine to that of selected disciplines which also influence the health system outside the tertiary setting, including the district health system (DHS) and the district clinical specialist teams. The ratios illustrate the significant maldistribution between public and private sectors. However, this model is flawed as it does not make sense to calculate targets for family physicians (FPs) in levels of care outside the DHS as FPs are not trained for the tertiary and quaternary levels of care. In 2014, a national position paper critiqued the incorrect underlying assumptions in the previous version of the HRH policy, which appeared to consider family medicine as a subspeciality of medicine in a referral hospital setting, rather than a generalist discipline in the DHS.^5^ How is it possible that this erroneous assumption seems to persist despite the growing evidentiary basis on the contribution of FPs in the South African DHS?^6^

**TABLE 1:** Actual and target ratios of selected specialities per 100 000 population, 2019.

DCST speciality	Current 2019				Projected 2025	
	Public sector	Private sector	South Africa total	Recommended target	South Africa total	Recommended target
Family medicine	0.66	3.78	1.13	2.00	1.21	2.14
Anaesthesiology	0.64	9.69	2.03	5.00	2.48	5.23
Obstetrics and gynaecology	0.62	6.57	1.53	2.40	1.83	2.63
Paediatrics	0.89	4.34	1.42	4.00	1.83	4.05

*Source*: National Department of Health. 2030 human resources for health strategy: Investing in the health workforce for universal health coverage [homepage on the Internet]. Pretoria: Government Printers; 2020 [cited 2021 May 11]. Available from: https://www.spotlightnsp.co.za/wp-content/uploads/2020/08/2030-HRH-strategy-19-3-2020.pdf

DCST, district clinical specialist team.

The new HRH policy cited the experiences in Brazil, Ghana, Mexico and Thailand to support the value of investing in HRH to achieve Universal Health Coverage.^2^ Numerically speaking, it would therefore make sense to compare our FP supply with that of these countries. In 2015, South Africa had one FP per 100 000 population compared to two FPs per 100 000 in Brazil.^6^ In 2016, Ghana only had 36 trained FPs with 29 residents in training.^7^ In 2017, Mexico had 30 FPs per 100 000 and Thailand had 9 FPs per 100 000.^7^ Previously, the World Bank experts have suggested an absolute minimum of 30 FPs per 100 000.^6^ A recent paper describes the supply of FPs in South Africa based on registered FPs from 2002 until 2019.^8^ It showed that a total of 969 specialist FPs were registered in 2019. If we were to accept the underlying assumptions of the new HRH policy’s models and their 2025 target as 2.14 FPs per 100 000, we require a total of 1275 FPs in 2025, which equals 306 additional FPs. To achieve this over the next 5 years, we need to produce 61 new FPs per year. At present, the training programmes produce 20 FPs per year. This means that nationally, we need to triple our output of graduates, which confirms the forecasting described in the FP HRH analysis paper.^8^ The implications for training posts and training programmes are considerable.

This editorial is a mere introduction to the new HRH policy and should by no means be viewed as a comprehensive assessment. In addition, this new policy was drafted in a pre-COVID-19 world. Fortunately, the goals of our South African Academy of Family Physicians (SAAFP) Council include influencing the new national HRH policy.^9^ Several platforms are available for the active engagement of the SAAFP community, including this journal and our national conference.^10^ Let us:

[*S*]how our clinical and health planning colleagues, as well as our politicians, that building the future with family doctors is the right approach, at the right time, to achieve our shared goal of comprehensive patient-centred health services.^1^

Best wishes,

Klaus B. von Pressentin

Editor-in-Chief

## References

[1] World Organisation of Family Doctors. World Family Doctor Day [homepage on the Internet]. 2021 [cited 2021 May 11]. Available from: https://www.globalfamilydoctor.com/member/ForMemberOrganizations/WorldFamilyDoctorDay.aspx

[2] National Department of Health. 2030 human resources for health strategy: Investing in the health workforce for universal health coverage [homepage on the Internet]. Pretoria: Government Printers; 2020 [cited 2021 May 11]. Available from: https://www.spotlightnsp.co.za/wp-content/uploads/2020/08/2030-HRH-strategy-19-3-2020.pdf

[3] White KL, Williams TF, Greenberg BG. The ecology of medical care. Bull N Y Acad Med. 1961;73(1):187. 10.1056/NEJM196111022651805 PMC2359390 8804749

[4] World Health Organization. Primary health care systems (PRIMASYS): Case study from South Africa [homepage on the Internet]. World Health Organization; 2017 [cited 2021 May 11]. Available from: https://www.who.int/alliance-hpsr/projects/alliancehpsr_southafricaprimasys.pdf?ua=1

[5] Mash R, Ogunbanjo G, Naidoo SS, Hellenberg D. The contribution of family physicians to district health services: A national position paper for South Africa. S Afr Fam Prac. 2015;57(3):54–61.

[6] Mash R, Von Pressentin KB. Strengthening the district health system through family physicians. S Afr Health Rev. 2018;2018(1):33–39.

[7] Wilfrid Laurier University. Global family medicine: “Mapping” family medicine around the world [homepage on the Internet]. 2021 [cited 2021 May 11]. Available from: https://globalfamilymedicine.org/

[8] Tiwari R, Mash R, Karangwa I, Chikte U. A human resources for health analysis of registered family medicine specialists in South Africa: 2002–19. Fam Prac. 2021;38(2):88–94. 10.1093/fampra/cmaa084 32914851

[9] South African Academy of Family Physicians. Academy newsletter [homepage on the Internet]. 2021 [cited 2021 May 11]. Available from: https://saafp.org/2021/05/11/academy-newsletter-2/

[10] South African Academy of Family Physicians. SAAFP Conference 2021 [homepage on the Internet]. 2021 [cited 2021 May 11]. Available from: https://www.saafp2021.org.za/

